# Dr. Clayton M. Daniels

**Published:** 1902-10

**Authors:** 


### OBITUARY.

Dr. Clayton M. Daniels, of Buffalo, died at his home, 315 Jersey Street, September 6, 1902, aged 48 years. It is believed by his intimate friends that he was infected through an operation wound a long time ago, though he never mentioned the fact. At all events, he has been ill for many months, confined to his bed most of the time, with symptoms that would justify such a conclusion.

Clayton M. Daniels was born at Panama, N.Y., April 2, 1854. He was educated in the public schools of that village and was afterward graduated from the Jamestown High School. He then entered the college of medicine of the University of Buffalo, was graduated in 1880 and began the practice of medicine and surgery in Buffalo. His chief work for many years had been of a surgical nature, having early developed a preference in that direction, and he became a deft operator as well as a skilful surgeon.

At the time of his death Dr. Daniels was surgeon-in-chief of the Sisters of Charity and the Emergency Hospitals and had been for years back. He selected the site, and bought the lot for the new Emergency hospital and made the general draft of the plans for that building, in which he took great pride.

For four years Dr. Daniels was professor of anatomy in the medical college of Niagara University. He was also attending surgeon at Buffalo for the Erie, Nickel Plate, Western New York & Pennsylvania and Buffalo, Rochester & Pittsburg railroads.

He was a prominent Mason and that brotherhood conducted the funeral ceremonies on Monday afternoon, September 8, according to the ritual for the burial of the dead of that order, the interment being at Forest Lawn Cemetery.

Dr. Daniels married Elva T. Ellis twenty-seven years ago. He is survived by the widow and by one daughter, Laura E. Daniels.

